# Supplementary material for: Artificial spider silk from ion-doped and twisted core-sheath hydrogel fibres
Source: Nat Commun. 2019 Nov 22;10:5293. doi: 10.1038/s41467-019-13257-4 (PMC6874677; doi:10.1038/s41467-019-13257-4)
Supplement: Supplementary file 1 — Supplementary Information [file 41467_2019_13257_MOESM1_ESM.pdf]

Supplementary Information

Ion-Doped and Twisted Core-Sheath Hydrogel Fibres with Spider Silk-Like Strength, Toughness, and Stretchability

Yuanyuan Dou[1], Zhen-Pei Wang[2], Wenqian He[1], Tianjiao Jia[1], Zhuangjian Liu[2], Pingchuan Sun[1], Kai Wen[1,3], Enlai Gao[4], Xiang Zhou[3], Xiaoyu Hu[1], Jingjing Li[1], Shaoli Fang[5], Dong Qian[6], Zunfeng Liu[1]*

[1]State Key Laboratory of Medicinal Chemical Biology, College of Pharmacy, Key Laboratory of Functional Polymer Materials, Nankai University, Tianjin 300071, China
[2]Institute of High Performance Computing, A*STAR Research Entities, Singapore 138632, Singapore
[3]Department of Science, China Pharmaceutical University, Nanjing, Jiangsu, 211198, China
[4]Department of Engineering Mechanics, School of Civil Engineering, Wuhan University, Wuhan, Hubei 430072, China
[5]Alan G. MacDiarmid NanoTech Institute, University of Texas at Dallas, Richardson, TX 75080, USA
[6]Department of Mechanical Engineering, University of Texas at Dallas, Richardson, TX 75080, USA

*To whom correspondence should be addressed. E-mail: liuzunfeng@nankai.edu.cn

This PDF file includes the following:

Supplementary Notes 1 to 5, Supplementary Figures 1 to 4 (Pages S2–S8)
Supplementary Tables 1 to 7 (Pages S9–S14)
Supplementary Figures 5 to 18 (Pages S15–S24)
Supplementary Videos 1 to 4 (Page S25)
Supplementary References (Pages S26–28)

Supplementary Note 1. Characterisation Methods

The SEM images were taken on a scanning electron microscopy (FEI Quanta 200). Fourier transform infrared (FTIR) spectroscopy was performed using a TENSOR 37 FTIR spectrometer. Confocal optical images and diameter of hydrogel fibres were acquired using a laser confocal microscope (Leica TCS SP8). Optical images of hydrogel fibers were also obtained on a metallographic microscope in a reflective mode (Chenxing CXML1000). Ambient temperature and relative humidity were measured on a hygrometer (CEM DT-615). Solid-state nuclear magnetic resonance (NMR) spectra were obtained on a Varian Infinity-plus 400 spectrometer at 55 kHz and 40 kHz fast magic-angle-spinning (MAS). Mechanical properties of the hydrogel fibres were measured using an Instron mechanical tester (model 3365). The specimens for mechanical test were taped to paper frames using double-sided adhesive tape, with a gauge length of 30 mm. The frames were installed on the Instron tester equipped with a calibrated 5 N load cell. The deformation rate was 20–500 mm min^{-1} (corresponding to 1.1–27.8% s^{-1}).

Supplementary Note 2. Calculations of toughness, energy dissipation, damping capacity, and degree of supercontraction

Tensile stress (σ) and tensile strain (ε) are engineering values. Tensile stress was calculated as:

$$\sigma = T/\pi r^2 \quad (1)$$

where T is the traction load and r is the initial radius of the hydrogel fibre.

Tensile strain (ε) was calculated as the ratio of length change (l–l_0) with the initial length (l_0) of the hydrogel fibre, where l is the stretched length:

$$\varepsilon = (l - l_0)/l_0 \times 100\% \quad (2)$$

Toughness (E) was calculated by integrating the area under the stress−strain curve before fibre fracture:

$$E = \int_0^{\varepsilon} \sigma d\varepsilon \quad (3)$$

It was subsequently normalized to the fibre volume (V) or mass (m) to give the volumetric toughness (E_{vol}) or gravimetric toughness (E_m), respectively:

$$E_{vol} = E/V \quad (4)$$

$$E_m = E/m \quad (5)$$

Energy dissipation (ΔE) for hydrogel fibres was calculated using the area of the hysteresis loop from the stress-strain curves with loading and unloading processes:

$$\Delta E = \int_{\text{loading}} \sigma d\varepsilon - \int_{\text{unloading}} \sigma d\varepsilon \quad (6)$$

Damping capacity (η) of the hydrogel fibre during the loading-unloading processes was evaluated as:

$$\eta = \Delta E/E \times 100\% \quad (7)$$

Supercontraction of the hydrogel fibre was recorded by a digital video camera during humidity changes in a closed chamber or being exposed to water vapour generated by ultrasonification in open air (40% RH, 25°C). Unless otherwise indicated, supercontraction was achieved by exposing a hydrogel fibre to water vapour and stopped by removing the vapour.

Degree of supercontraction (*DS*) was calculated using equation (8):

$$DS\ (\%) = l_s/l_0\times 100\% = (l-l_0)/l_0/100\% \qquad (8)$$

where Δl, l_0, l_s, are the length change during supercontraction, and the lengths of the hydrogel fibre before and during supercontraction, respectively.

Supplementary Note 3. Kinematic and kinetic analyses of a free falling weight connected to a hydrogel yarn

3.1 Equations of velocity, acceleration and impact force of a free falling weight connected to a hydrogel yarn

The deceleration process of the attached weight was recorded using a high-speed camera to obtain the velocity, acceleration and impact force of the weight. The velocity (v_o) of the weight can be calculated as:

$$v_o = dS/dt \qquad (9)$$

where *S* and *t* are the displacement and time of the falling weight, respectively. According to Newton's second law, the acceleration of the weight (*a*) is defined as:

$$a = dv_o/dt \qquad (10)$$

The weight applied an impact force (*F*) on the hydrogel yarn and was subjected to its own gravity during falling. According to Newton's second law of motion, the impact force on the yarn can be calculated as:

$$F = m_o g + m_o a \qquad (11)$$

where m_o is the mass of the weight and g is the gravitational acceleration.

3.2 Calculation of velocity, acceleration and impact force during shock-absorbing experiments

3.2.1 Vertical configuration

Based on the unique combination of high strength, ductility, toughness, and damping capacity of the hydrogel fibres, we investigated their ability to dissipate impact force for shock-absorbing applications. A 20-g weight was tethered to one end of a 10-cm-long hydrogel yarn comprising 100-ply, 20-μm-diameter fibres and allowed to freely fall at 60% humidity (Supplementary Video 1). Each hydrogel fibre contains 0.1 wt% VSNPs and 20 mM $ZnCl_2$ with a twist density of 3 turns mm^{-1}.

The fall was recorded using a high-speed camera (Sony DSC-RS100 M3, 1000 fps) to obtain the speed and acceleration of the weight. The weight fell 15 cm in 4 s. The speed of the weight increased first from 0 to a maximum of 0.8 m s^{-1} in 0.16 s before decreasing to 0 in 0.1 s (Supplementary Fig. 1a). The acceleration of the weight initially decreased from 9.8 m s^{-2} to 0 in 0.16 s, changed direction, and rose to 8.5 m s^{-2} within 0.06 s in the opposite direction (Supplementary Fig. 1b). The impact force of the hydrogel yarn increased from 0 to 0.36 N in

the initial 0.22 s and decreased to 0.2 N in the following 0.42 s. During this process, the hydrogel yarn extended by 150%, and the maximum impact force was 0.36 N, corresponding to 1.8 times the weight.

As a control experiment, a 20-g weight was tethered to a 200-μm-diameter, 25-cm-long cotton yarn, (the stress-strain curve is shown in Supplementary Fig. 3a). The weight was released 15 cm from the bottom, which is the same height as in the case of the hydrogel yarn (Supplementary Video 1), and reached a maximum speed of 1.4 m s^{-1} (Supplementary Fig. 1c). During the fall, the cotton yarn elongated by 3% and incurred a maximum impact force of 3.1 N (Supplementary Fig. 1d), which is 8.6 times that of the hydrogel yarn. This indicates that the hydrogel fibre is an excellent candidate as an impact reduction material.

3.2.2 Horizontal configuration

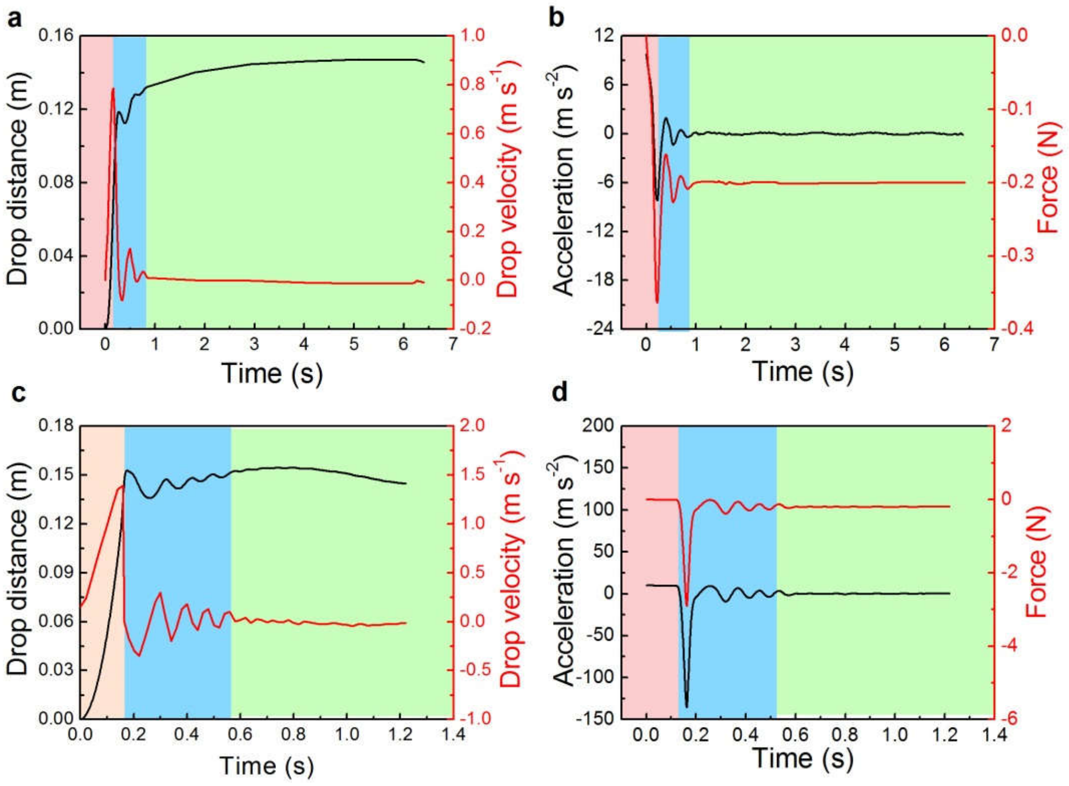

Supplementary Figure 1. (**a, c**) Displacement and velocity and (**b, d**) acceleration and impact force of a free-falling 20-g load tethered to a yarn as a function of time for the vertical configuration. The yarn in (**a, b**) was a 10-cm-long yarn composed of 100-ply, 20-μm-diameter hydrogel fibres, and the yarn in (**c, d**) was a 25-cm-long, 200 μm-diameter cotton yarn. The top ends of the hydrogel yarn and the cotton yarn were positioned at the same height and the load fell by 15 cm. Each hydrogel fibre contained 0.1 wt% VSNPs and 20 mM $ZnCl_2$ with a twist density of 3 turns mm^{-1} and a RH of 60%. The data were collected from Supplementary Video 1.

In order to investigate the impact reduction of the hydrogel fibre at a higher impaction speed, we used a horizontal configuration of hydrogel yarn. A 10-cm-long yarn comprising 100-

ply, 20-μm-diameter hydrogel fibres was horizontally tethered between two posts and a 20-g weight was attached to the middle of the hydrogel yarn using a 30-cm-long, 100-μm-diameter polyethylene fishing line (Supplementary Video 2). The weight was released 30 cm from the bottom. During the fall, the speed of the weight increased to maximum value of 2.4 m s^{-1} before decreasing (Supplementary Fig. 2), and the weight slightly bounced and then stopped (Supplementary Fig. 2a). The maximum impact force was 2.2 N, which is 11.5 times the load weight (Supplementary Fig. 2b).

When the hydrogel yarn was replaced with a 10-cm-long, 200-μm-diameter cotton yarn, the weight stopped abruptly and achieved a maximum impact force of 20 N, which is 9.1 times that when using the hydrogel yarn (Supplementary Fig. 2d). When the hydrogel yarn was replaced with a 10-cm-long, 1.5-mm-diameter natural rubber fibre (the stress-strain curve is shown in Supplementary Fig. 3b), the weight slowed down and bounced up and down many times before stopping (Supplementary Video 2), indicating high resilience and low damping capacity. The maximum impact force amounted to 10 N.

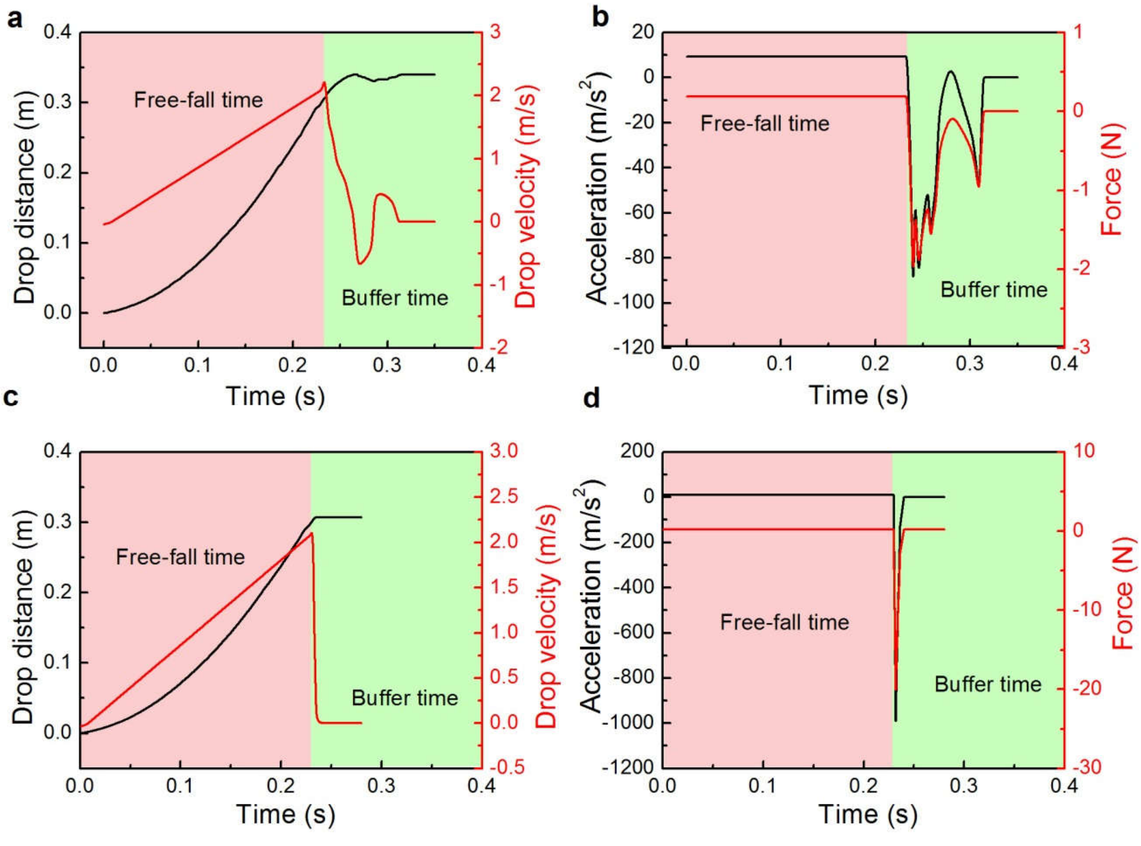

Supplementary Figure 2. (**a, c**) Displacement and velocity and (**b, d**) acceleration and impact force as a function of time for a free-falling 20-g load tethered to the middle of a horizontally positioned yarn via a 30-cm-long, 0.1-mm-diameter polyethylene fishing line. The yarn in (**a, b**) was a 10-cm-long yarn composed of 100-ply, 20-μm-diameter hydrogel fibres, and the yarn in (**c, d**) was a 10-cm-long, 200 μm-diameter cotton yarn. The load fell from the same height of 30 cm in both cases. Each hydrogel fibre contained 20 mM $ZnCl_2$ and presented a twist density of 3 turns mm^{-1} and the RH was 60%. The data were collected from Supplementary

Video 2.

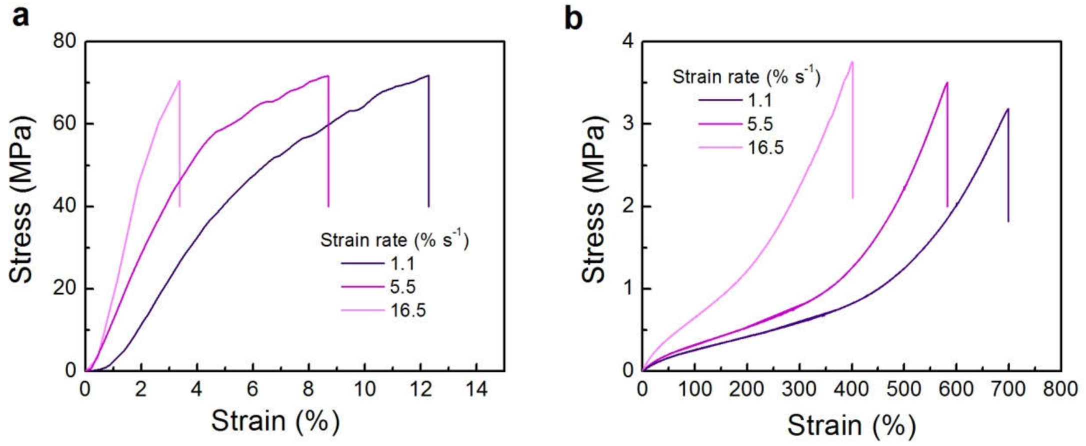

Supplementary Figure 3. Stress-strain curves of the cotton yarn (**a**) and the rubber fibre (**b**) with different strain rates. The diameters of the cotton yarn and rubber fibre were 200 μm and 1.5 mm, respectively.

Supplementary Note 4. Calculation of the toughness of the hydrogel yarn at different deformation rates in the horizontal configuration described in Section 3.2.2

The fall process of the yarn-tether weight was recorded using a high-speed camera to obtain the velocity of the weight. According to Newton's second law of motion, the gravitational potential energy change (G) of an object falling to a minimum height can be calculated using:

$$G = m_0 g h \quad (12)$$

where h is the falling distance of the object. The law of conservation of energy implies that the toughness of the hydrogel yarn during the object's fall is equal to the gravitational potential energy change. Therefore, the volumetric toughness for horizontal configuration (E_f) is calculated as:

$$E_f = G/V \quad (13)$$

Supplementary Note 5. FEM model of the core-sheath hydrogel fibres based stress-strain curve

From the optical microscope images in Fig. 1d, it can be seen that the hydrogel fibres have outer covering sheath, whose thickness increased with increasing the exposure time in low humidity environment. This essentially re-characterizes the mechanical properties of the fibres by means of the combination of a soft core with low Young's modulus and a stiff sheath with high Young's modulus, leading to fibres with the same diameter at different drying time exhibiting different mechanical properties. As the fibres were exposed in 40% humidity air for different time, we suppose that the mechanical properties of the fibre sheath and fibre core keeps constant and their dimensions change with time. So if we know the upper bound limit (fibre sheath) and the lower bound limit (fibre core) of the hydrogel fibre, we could fit the mechanical properties of the hydrogel fibres with different exposure time. By using the freshly-drawn hydrogel fibre with drying time of 0 h. as the lower bound limit (fibre core), and using

the fibre dried at 40% humidity for 2 h as an upper bound limit (which shows the same mechanical properties before the yielding point as the fibre dried for 4 h).

To elaborate this numerical fitting, a core-sheath model is proposed here by homogenizing the mechanical properties of the core and the sheath at certain conditions. With this model, the engineering tensile stress measure σ_j^i of the ith fibre satisfies the balance of force at strain state ε_j:

$$\sigma_j^i \pi {R^i}^2 = \sigma_{j,core}^i \pi (R_{core}^i)^2 + \sigma_{j,sheath}^i \pi [{R^i}^2 - (R_{core}^i)^2] \qquad (12)$$

where $\sigma_{j,core}^i$ and $\sigma_{j,sheath}^i$ represent the engineering tensile stress of the core and the sheath, respectively, R^i and R_{core}^i are the initial radius of the fibre and its core, respectively. Accordingly, the sheath thickness of the ith fibre is obtained as:

$$\gamma^i = R^i - R_{core}^i \qquad (13)$$

Henceforth, the sheath thickness γ^i of each fibre can be obtained inversely by minimizing a function

$$\Psi := \varphi_j^i \sum_i \sum_j \{\sigma_j^i {R^i}^2 - \sigma_{j,core}^i (R_{core}^i)^2 - \sigma_{j,sheath}^i [{R^i}^2 - (R_{core}^i)^2]\} \qquad (14)$$

where φ_j^i is the penalty factor for the ith fibre at strain ε_j. With these quantified sheath stiffness, detailed finite element model can be built to verify the experimental results and further numerically predict the mechanical performance of the fibres.

To verify the mechanical performance of hydrogel fibres, finite element method (FEM) is used for numerical simulations based on commercial FEM software ABAQUS. The fibres in the FEM model are 200 μm long with different sheath thicknesses. The general-purpose 8-node linear brick element, with reduced integration (C3D8R), is used. Axial loading is applied at one end and the other end is fixed at the axial and tangential directions. The stress and strain values in the sheath and the core are obtained from numerical simulation results, and subsequently processed for comparison with experimental results. It should be noted that the numerical predictions of the stress-strain relations of the sheath-core models agree well with the experimental data at large strain.

Twist insertion results in increase in mechanical strength and modules of the hydrogel fibres. A numerical sheath-core model based on FEM were carried out to validate the effect of twist insertion on the internal residual stress/strain in a core-sheath structured hydrogel fibre. A 20-μm-diameter hydrogel fibre (sheath thickness of 3 μm, and core radius of 7 μm) was constructed in FEM. It is found that upon twist insertion (6 turns/mm), periodic internal stress along the fibre length was generated in both fibre sheath and fibre core, resulting in twisting of the hydrogel fibre and flattening in some regions (Supplementary Fig. S4b). This agrees with the experimental observation of twist-induced flattening of hydrogel fibres in Supplementary Fig. S4a.

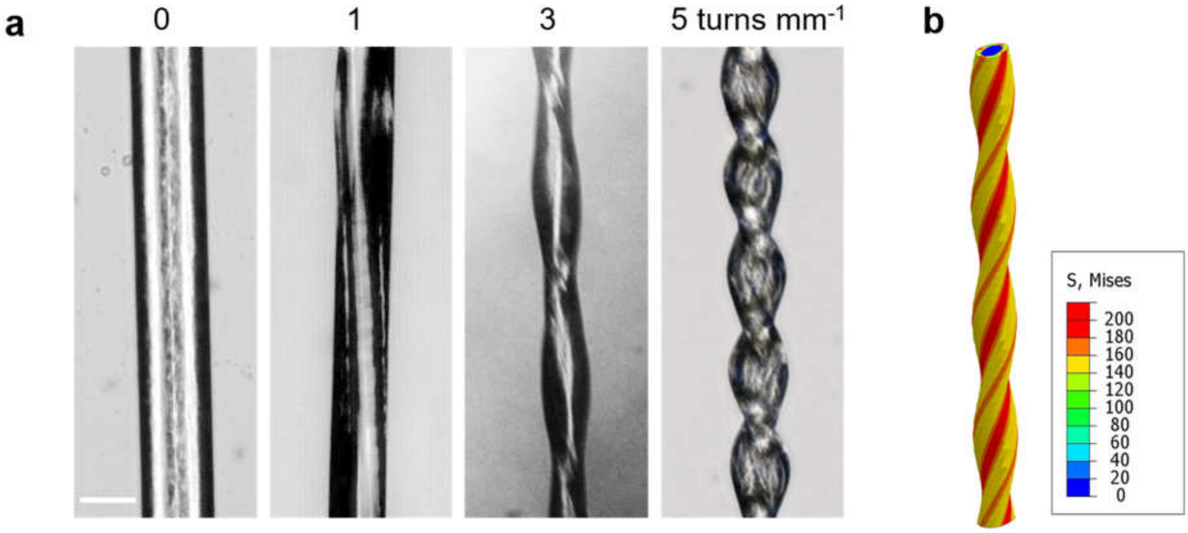

Supplementary Figure 4. (**a**) Metallographic microscopy images of the hydrogel fibres with different inserted twist. Scale bar: 40 μm. (**b**) FEM modelling showing stress distribution on the sheath of a 20-μm-diameter core-sheath hydrogel fibre. The inserted twist is 6 turns/mm, the sheath thickness is 3 μm, and core radius is 7 μm in FEM modelling.

Supplementary Tables

Supplementary Table 1. Comparison of the mechanical properties of typical artificial spider silks reported in the literatures

Artificial spider silk	Stress (MPa)	Strain (%)	Young's Modulus (GPa)	Fracture Energy (MJ m^{-3})	Ref.
Protein-Based Fibres	115	37	2.4	34	1
	150	84.5	–	89.05	2
	162	42	6.8	45	3
	169	3.35	6.64	3.66	4
	308	30	9.3	–	5
	320	100	8.0	–	6
	320	27	7.7	55	7
	325	27.3	5.2	60.2	8
	370	110	4.0	189	9
	383	95	5.0	172	10
	390	32.1	15.2	80.8	11
	450	27.7	–	0.055	12
	450	27.3	18.9	91	13
	570	24.5	8.6	103	14
	639	40	9.9	–	15
	895	59.6	42.8	–	16
	1345	36	13.44	334	17
Supramolecular Hydrogel Fibres	193	18	8.9	22.8	18
CNT Composite Fibres	120	150	0.032	–	19
	210	240	–	75	20
CNT/protein Composite Fibres	200	8	10	–	21
	600	73	–	290	22

Supplementary Table 2. Mechanical properties of as-prepared hydrogel fibres with different feed ratios

**Hydrogel fibre sample ($PAA/VSNPs_y/X_z$)**	**Breaking Strain (%)**	**Breaking stress (MPa)**	**Fracture Toughness ($MJ\ m^{-3}$)**	**Energy dissipation ($MJ\ m^{-3}$)**
$PAA/VSNPs_{0.1}$	73.1	127	53.3	50.5
$PAA/VSNPs_{0.2}$	60.8	150	50.5	47.8
$PAA/VSNPs_{0.3}$	49.4	186	47.9	45.5
$PAA/VSNPs_{0.4}$	40.0	197	44.6	42.3
$PAA/VSNPs_{0.5}$	10.1	207	14.3	13.6
$PAA/VSNPs_{0.1}/ZnCl_{2-0.01}$	53.0	226	85.5	81.0
**$PAA/VSNPs_{0.1}/ZnCl_{2-0.02}$**	**49.2**	**261**	**87.1**	**82.6**
$PAA/VSNPs_{0.1}/ZnCl_{2-0.03}$	59.2	218	85.7	81.2
$PAA/VSNPs_{0.1}/ZnCl_{2-0.04}$	66.4	181	83.8	79.4
$PAA/VSNPs_{0.1}/ZnCl_{2-0.06}$	71.0	145	75.6	71.7
$PAA/VSNPs_{0.2}/ZnCl_{2-0.02}$	9.0	253	21.3	20.2
$PAA/VSNPs_{0.3}/ZnCl_{2-0.02}$	6.2	289	16.4	15.5
$PAA/VSNPs_{0.4}/ZnCl_{2-0.02}$	2.6	359	6.3	6.0
$PAA/VSNPs_{0.1}/MgCl_{2-0.02}$	55.0	150	75.1	71.1
$PAA/VSNPs_{0.1}/NaCl_{0.02}$	59.8	145	66.3	62.8
$PAA/VSNPs_{0.1}/KCl_{0.02}$	71.8	134	54.5	51.8

$PAA/VSNPs_y/X_z$: X refers to the type of metal chloride salt; y refers to the weight ratio of VSNPs in hydrogel; z refers to the molar concentration (M) of salt in hydrogel. The same notation is used in Supplementary Tab. 3 and 4. The mechanical properties of the hydrogel fibres were measured at 40% RH with a deformation rate of 1.1% s^{-1}.

Supplementary Table 3. Mechanical properties of hydrogel fibres at various relative humidity, deformation rates, and inserted twists

**Hydrogel fibre sample ($PAA/VSNPs_y/X_Z$)**	**Condition**	**Breaking stress (MPa)**	**Breaking strain (%)**	**Toughness ($MJ\ m^{-3}$)**	**Energy dissipation ($MJ\ m^{-3}$)**
$PAA/VSNPs_{0.1}/ZnCl_{2-0.02}$	30% RH[(a)]	264	48.1	86.5	82.0
**$PAA/VSNPs_{0.1}/ZnCl_{2-0.02}$**	**40% RH[(a)]**	**261**	**49.2**	**87.2**	**82.6**
$PAA/VSNPs_{0.1}/ZnCl_{2-0.02}$	50% RH[(a)]	217	59.5	79.3	72.9
$PAA/VSNPs_{0.1}/ZnCl_{2-0.02}$	60% RH[(a)]	192	86.2	84.0	74.7
$PAA/VSNPs_{0.1}/ZnCl_{2-0.02}$	70% RH[(a)]	169	100	72.3	60.1
$PAA/VSNPs_{0.1}/ZnCl_{2-0.02}$	80% RH[(a)]	133	124	52.1	36.9
$PAA/VSNPs_{0.1}/ZnCl_{2-0.02}$	100%RH[(a)]	82.3	159	51.4	26.7
$PAA/VSNPs_{0.1}/ZnCl_{2-0.02}$	0.6% s^{-1}[(b)]	213	49.3	65.1	61.7
$PAA/VSNPs_{0.1}/ZnCl_{2-0.02}$	1.1% s^{-1}[(b)]	261	49.2	87.2	82.6
$PAA/VSNPs_{0.1}/ZnCl_{2-0.02}$	2.8% s^{-1}[(b)]	316	49.3	104	99.2
$PAA/VSNPs_{0.1}/ZnCl_{2-0.02}$	4.4% s^{-1}[(b)]	367	49.3	123	116
$PAA/VSNPs_{0.1}/ZnCl_{2-0.02}$	5.6% s^{-1}[(b)]	418	49.3	149	141
$PAA/VSNPs_{0.1}/ZnCl_{2-0.02}$	11.1% s^{-1}[(b)]	468	49.2	176	167
$PAA/VSNPs_{0.1}/ZnCl_{2-0.02}$	27.8% s^{-1}[(b)]	514	49.3	210	199
$PAA/VSNPs_{0.1}/ZnCl_{2-0.02}$	1 turns mm^{-1}[(c)]	593	58.3	157	149
**$PAA/VSNPs_{0.1}/ZnCl_{2-0.02}$**	**3 turns mm^{-1}[(c)]**	**647**	**67.4**	**259**	**245**
$PAA/VSNPs_{0.1}/ZnCl_{2-0.02}$	5 turns mm^{-1}[(c)]	684	53.3	216	205
**$PAA/VSNPs_{0.1}/ZnCl_{2-0.02}$**	**7 turns mm^{-1}[(c)]**	**895**	**44.3**	**214**	**201**
$PAA/VSNPs_{0.1}/ZnCl_{2-0.02}$	1 turns mm^{-1}[(d)]	280	61.2	129	122
$PAA/VSNPs_{0.1}/ZnCl_{2-0.02}$	3 turns mm^{-1}[(d)]	369	68.5	221	205
$PAA/VSNPs_{0.1}/ZnCl_{2-0.02}$	5 turns mm^{-1}[(d)]	427	56.9	142	132
$PAA/VSNPs_{0.1}/ZnCl_{2-0.02}$	7 turns mm^{-1}[(d)]	640	47.1	195	183

To study the influence of one parameter on the mechanical properties, other parameters were optimised step by step. For (a), the deformation rate was 1.1% s^{-1}, with no inserted twist; for (b), the RH was 40%, with no inserted twist; for (c), the RH was 40%, and the deformation rate was 27.8% s^{-1}; for (d), the RH was 40%, the deformation rate was 27.8% s^{-1}, and the sample was a self-balanced two-ply fibre.

Supplementary Table 4. Mechanical properties of hydrogel fibres presenting 3 turns mm^{-1} twists at different relative humidity and deformation rates

**Hydrogel fibre sample ($PAA/VSNPs_y/X_Z$)**	**Condition**	**Breaking stress (MPa)**	**Breaking strain (%)**	**Toughness (MJ m^{-3})**	**Energy dissipation (MJ m^{-3})**
$PAA/VSNPs_{0.1}/ZnCl_{2-0.02}$	0.6% s^{-1} (a)	271	67.4	80.5	76.3
$PAA/VSNPs_{0.1}/ZnCl_{2-0.02}$	1.1% s^{-1} (a)	332	67.3	108	102
$PAA/VSNPs_{0.1}/ZnCl_{2-0.02}$	2.8% s^{-1} (a)	398	67.2	130	123
$PAA/VSNPs_{0.1}/ZnCl_{2-0.02}$	4.4% s^{-1} (a)	462	67.3	151	143
$PAA/VSNPs_{0.1}/ZnCl_{2-0.02}$	5.6% s^{-1} (a)	527	67.3	183	174
$PAA/VSNPs_{0.1}/ZnCl_{2-0.02}$	11.1% s^{-1} (a)	589	67.4	217	205
**$PAA/VSNPs_{0.1}/ZnCl_{2-0.02}$**	**27.8% s^{-1} (a)**	**647**	**67.4**	**259**	**245**
**$PAA/VSNPs_{0.1}/ZnCl_{2-0.02}$**	10-ply(b)	614	66.5	241	226
**$PAA/VSNPs_{0.1}/ZnCl_{2-0.02}$**	30-ply(b)	593	56	219	208
**$PAA/VSNPs_{0.1}/ZnCl_{2-0.02}$**	50-ply(b)	554	46	196	182
**$PAA/VSNPs_{0.1}/ZnCl_{2-0.02}$**	100-ply(b)	451	38	147	136
$PAA/VSNPs_{0.1}/ZnCl_{2-0.02}$	30% RH(b)	648	67.3	256	243
**$PAA/VSNPs_{0.1}/ZnCl_{2-0.02}$**	**40% RH(b)**	**647**	**67.4**	**258**	**245**
$PAA/VSNPs_{0.1}/ZnCl_{2-0.02}$	50% RH(b)	512	81.3	235	217
$PAA/VSNPs_{0.1}/ZnCl_{2-0.02}$	60% RH(b)	453	117	249	222
$PAA/VSNPs_{0.1}/ZnCl_{2-0.02}$	70% RH(b)	399	137	215	172
$PAA/VSNPs_{0.1}/ZnCl_{2-0.02}$	80% RH(b)	315	160	155	110
$PAA/VSNPs_{0.1}/ZnCl_{2-0.02}$	100%RH(b)	194	207	153	79.6

For (a), the RH was 40% and the twist density was 3 turns mm^{-1}; for (b), the deformation rate was 27.8% s^{-1} and the twist density was 3 turns mm^{-1}.

Supplementary Table 5. Comparison of the mechanical properties of hydrogel fibres, spider silk fibres and other synthetic fibres

Materials	Toughness (MJ m^{-3})	Breaking stress (MPa)	Breaking strain (%)	Initial modulus (GPa)	Energy dissipation (MJ m^{-3})	Damping capacity (%)	Ref.
hydrogel fibre	259	647	67.4	12.9	245	94.8	This work
hydrogel fibre	225	895	44.3	28.7	214	94.8	This work
Araneus MA silk	160	1,100	27	10	104	65	44
Araneus viscid silk	150	500	270	0.003	97.5	65	44
Nylon 6.6	80	750	18	5.0	32	40	43
Kevlar 49	50	3,000	2.7	130	–	–	43
Wool	60	200	5	0.5	39.6	66	43
Tendon collagen	7.5	150	1.5	12	0.53	7	43
Elastin	2	1	150	0.001	0.2	10	43
Carbon fibre	25	4,000	1.3	300			43
Resilin	4	3	190	2	0.24	6	43
High-tensile steel	6	1,650	0.8	200	–	–	43
Synthetic rubber	100	50	850	0.001	–	–	44

MA silk denotes the silk from the major ampullate gland.

Supplementary Table 6. Comparison of the mechanical properties of the hydrogel fibres obtained in this work with state-of-the-art low-density compressible foams and hydrogel materials, reported in literatures

Materials	Toughness (MJ m^{-3})	Compressive or tensile stress (MPa)	Energy dissipation (MJ m^{-3})	Breaking strain (%)	Damping capacity (%) (strain)	Ref.
hydrogel Fibres	259	647	245	67.4	0.948	This work
hydrogel Fibres	225	895	214	44.3	0.948	This work
CNT Sponge	2.4	5	2.4	−80	0.8	24
CNT Array	0.283	1.6	0.283	−70	0.64–0.42	32
Carbon-Graphene Monoliths	0.067	0.025	0.0024	−80	0.36–0.26	25
CNT Films	11.3	26	7.29	−85	0.64–0.47	31
Graphene Aerogels	0.298	1.2	0.145	−50	0.60–0.31	30
Graphene Monoliths	0.006	0.018	0.015	−80	0.87–0.67	27
Poly(acrylamide-co-acrylic acid) Hydrogels	14	4.5	12.2	500	0.87	28
Nano-composite Hydrogels	6.25	0.15	3.5	8,000	0.56	29
Toughening Elastomers	7.86	13.5	7	72	0.89	23
SBR 05 Rubbers	0.16	3.8	0.13	179	0.78	26
Polyacrylamide-Chitosan Hydrogels	3.78	2.5	3.26	300	0.86	38

Supplementary Table 7. Toughness of the hydrogel yarn at different deformation rates in a horizontal configuration (as in Section 3.2.2)

Height (m)	Deformation rate (% s^{-1})	Toughness (MJ m^{-3})
0.1	1,400	4.6
0.3	2,400	13.5
0.5	3,100	22.6
0.7	3,700	32.2
1.0	4,400	46.1
3.0	7,600	138.4
5.0	9,800	230.7
7.0	11,000	321.1
8.0	12,000	369.7

Supplementary Figures

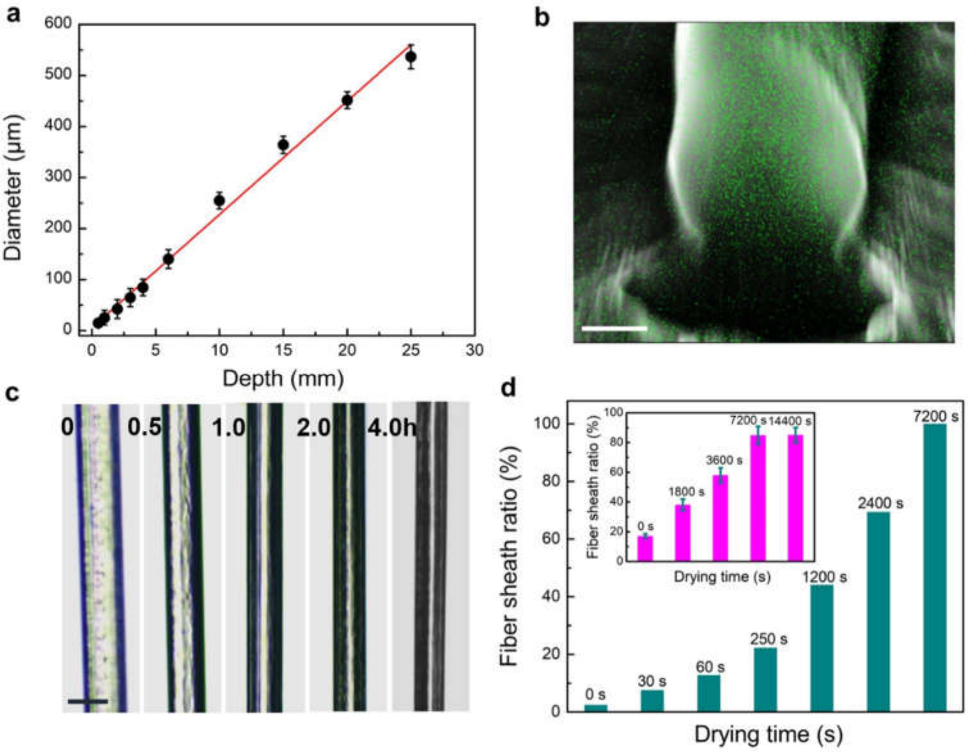

Supplementary Figure 5. (**a**) Dependence of the hydrogel fibre diameter on the immersion depth of the fibre drawing steel rod in the hydrogel reservoir. (**b**) Elemental mapping shows that Zn^{2+} ions uniformly distributed in the cross-section of the fibre. Scale bar: 2 μm. (**c**) Metallographic microscopy images of the hydrogel fibre exposed in ambient air (40% humidity) for different time in a reflective mode. Scale bar: 50 μm. (**d**) Optically-measured (inset) and theoretically calculated fiber sheath ratio.

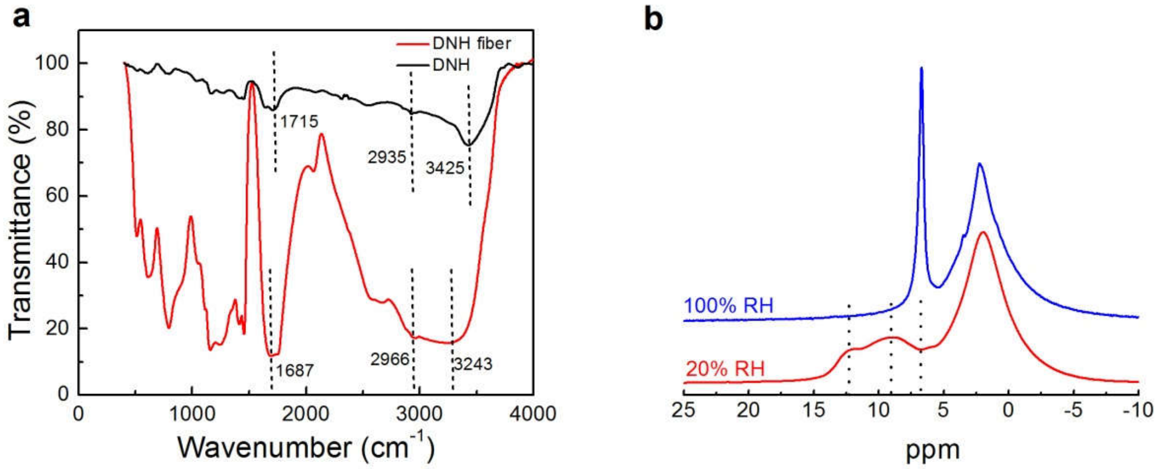

Supplementary Figure 6. (**a**) FTIR spectra of the hydrogel gel and fibres. The absorption bands at 1715, 2935, and 3425 cm^{-1} in the gel are attributed to the carboxyl (–COOH), vinyl (=CH–CH_2), and hydroxyl groups (–OH), respectively. For hydrogel fibres, the absorption bands of the carboxyl and hydroxyl groups shift to 1687 cm^{-1} and 3243 cm^{-1}, respectively. This indicates that hydrogel fibres exhibit more hydrogen bonding between macromolecular chains than the parent gel. (**b**) Solid-state NMR spectra at 55 kHz fast MAS of the hydrogel fibre at two different relative humidity values. The spectra showed that three types of hydrogen bonds formed in hydrogel fibres at 20% RH. When water permeated the hydrogel fibres, hydrogen bonding decreased between polymer –COOH side groups while the competitive hydrogen bonding of water molecules increased.

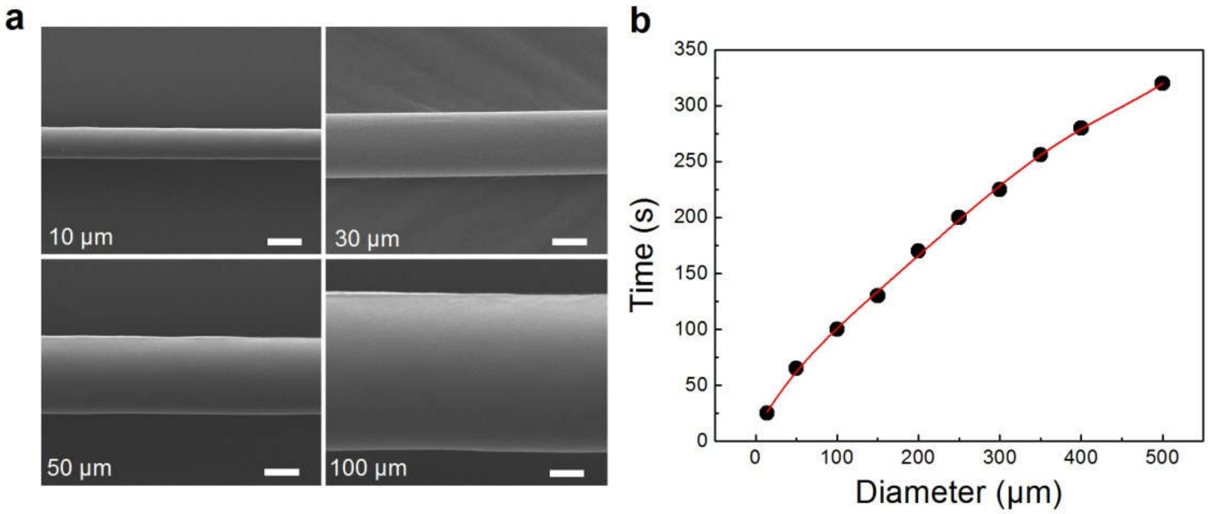

Supplementary Figure 7. (**a**) SEM images of hydrogel fibres displaying different diameters of 10, 30, 50, and 100 μm. The hydrogel fibres contained 0.1 wt% VSNPs, without inserted twist or salt, and the humidity was 40%. Scale bars: 10 μm. (**b**) Setting time (required minimum drying time) of hydrogel fibre as a function of as-drawn fibre diameter. This "setting time" was experimentally measured by mounting the as-stretched hydrogel fibre on a homemade holder, and exposing it in an ambient air of 40% humidity. The time that the fibre length did not change when releasing one end of the fibre was determined as the "setting time".

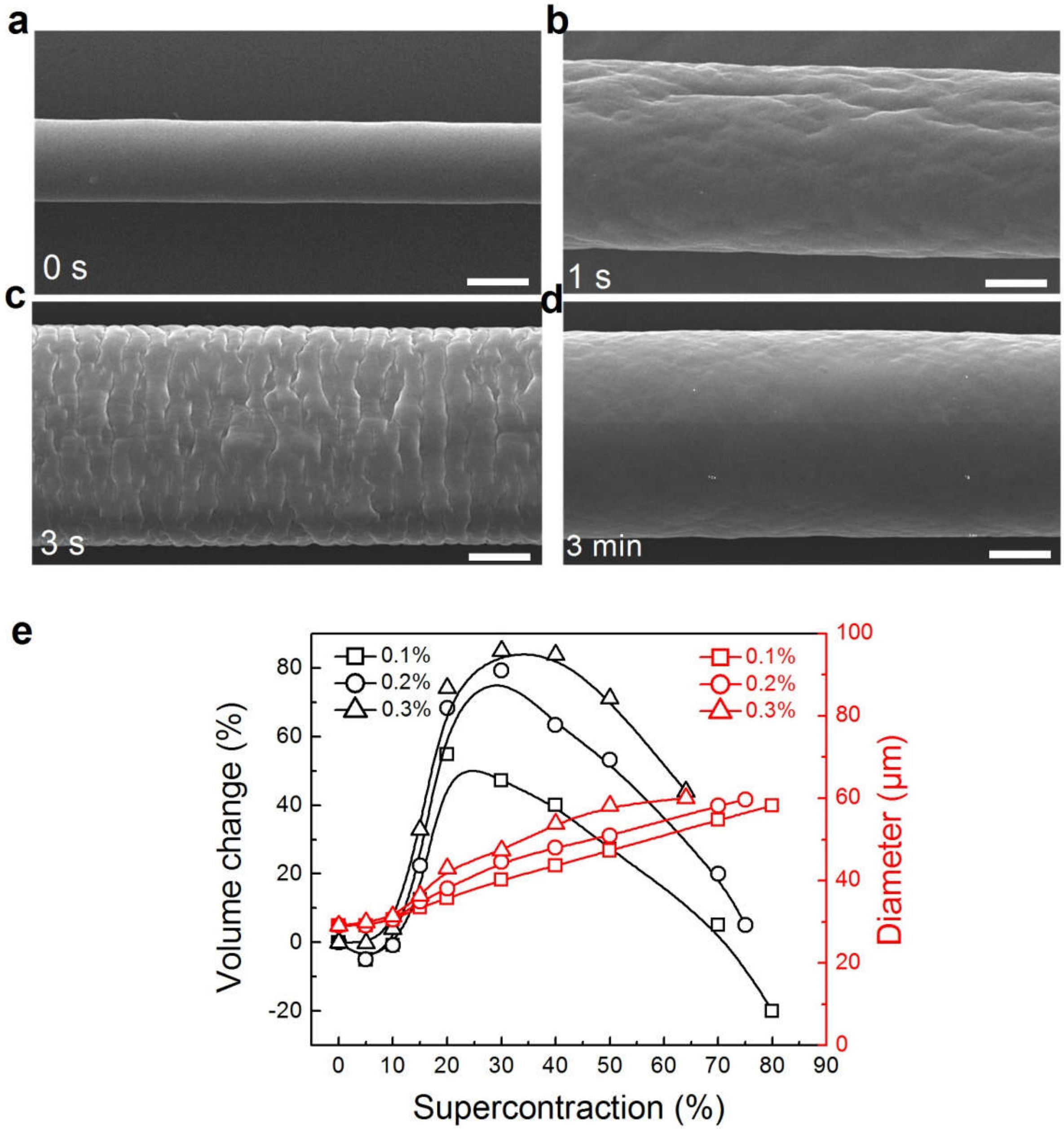

Supplementary Figure 8. SEM images of the hydrogel fibres just after (**a**) 0%, (**b**) 50%, and (**c**) 80% supercontraction as well as (**d**) three minutes after 80% supercontraction, upon exposure to 100% humidity. Scale bars: 20 μm. The hydrogel fibres contained 0.1 wt% VSNPs, without inserted twist or salt. (**e**) Volume and diameter changes with respect to the degree of supercontraction for hydrogel fibres with different VSNP contents, without inserted twist or salt. The hydrogel fibre was isobarically loaded with 0.18 MPa during supercontraction.

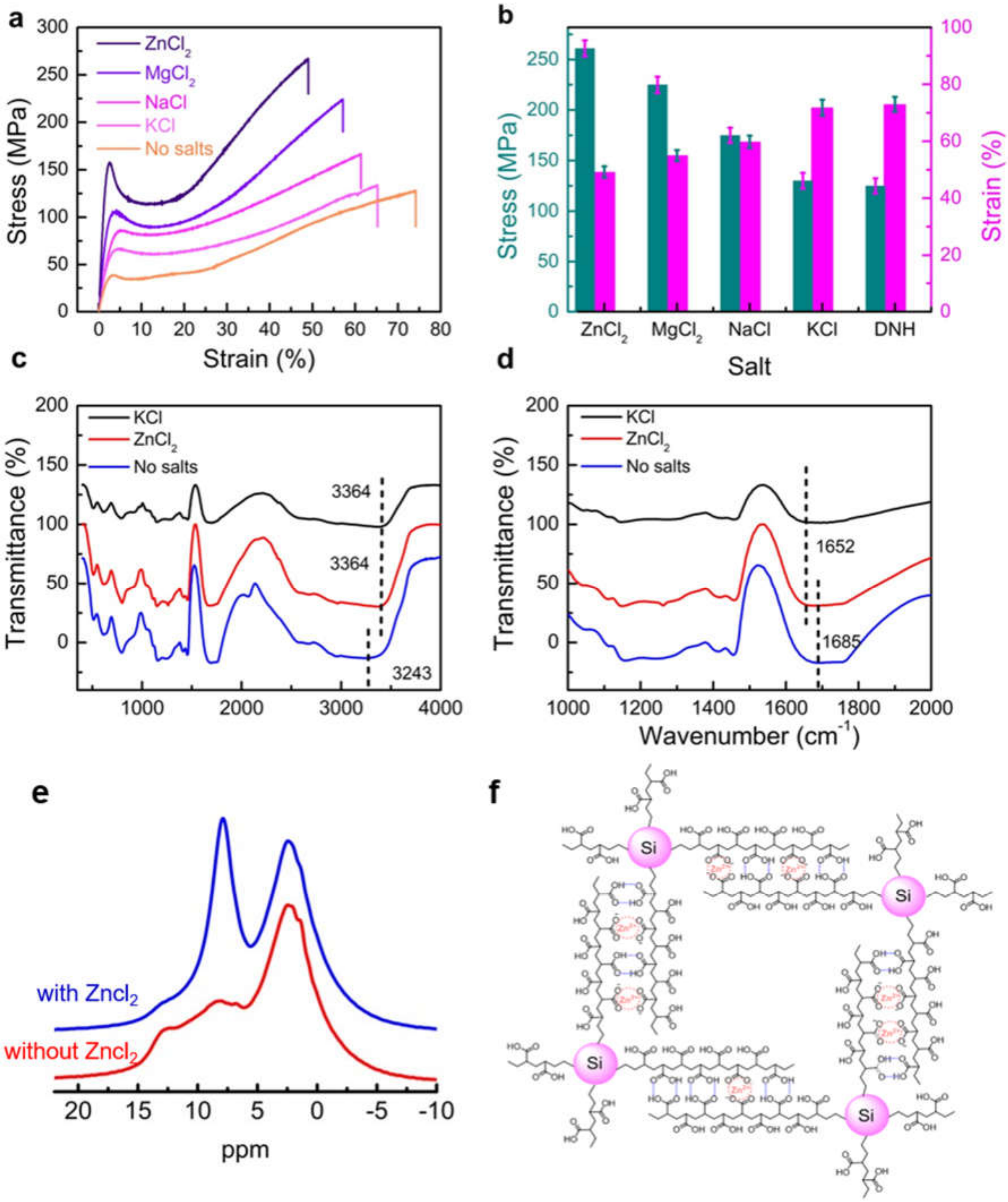

Supplementary Figure 9. (**a**) Tensile stress-strain curves and (**b**) stress and strain values for hydrogel fibres with different types of salts. The hydrogel fibres contained 0.1 wt% VSNPs and 20 mM salt, without inserted twist, and the diameter of fibres was 20 μm. The deformation rate was 1.1% s^{-1}. (**c, d**) FTIR spectra of hydrogel fibres containing KCl (20 mM) and $ZnCl_2$ (20 mM). Upon salt addition, the absorption band of the hydroxyl group up shifts from 3243 to 3364 cm^{-1} and that of the carboxyl group shifts from 1685 to 1652 cm^{-1}. This shows that the metal ions selectively bind to the carboxylic groups, which reduces hydrogen bonding between polymer chains. (**e**) ^{1}H MAS spectra at 40 kHz MAS of samples without and with $ZnCl_2$. (**f**) Schematic demonstration of physical cross-linking of hydrogen bonding of polyacrylic chains

and ionic cross-linking by Zn^{2+}, in addition to the covalent network by vinyl functionalized silica nanoparticles.

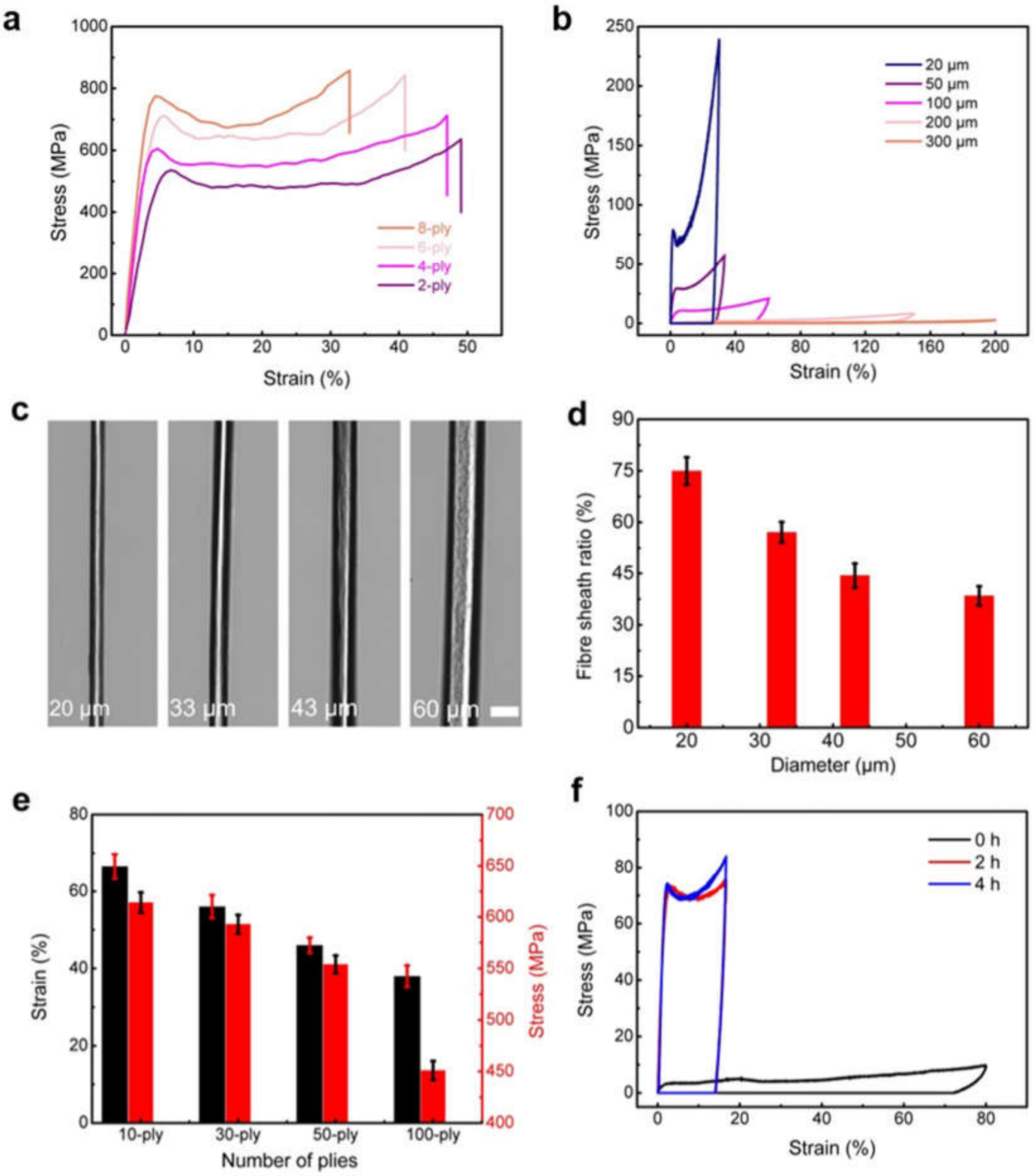

Supplementary Figure 10. (**a**) Tensile stress-strain curves of twisted hydrogel fibres presenting different number of plies for the self-balanced fibres. The hydrogel fibres contained 0.1 wt% VSNPs and 20 mM $ZnCl_2$, with 7 turns mm^{-1} of inserted twist. The deformation rate was 27.8% s^{-1}. (**b**) Loading-unloading curves of hydrogel fibres presenting different diameters. (**c**) Laser confocal microscope images of hydrogel fibres with different diameters. Scale bar: 40 μm. (**d**) Fibre sheath ratio of hydrogel fibres as a function of fibre diameter. (**e**) Breaking stress and breaking strain for different number of plies of hydrogel fibres. (**f**) Stress-strain curves for of 50-μm-diameter fibres with different drying time. The black, red and blue curves are for hydrogel fibres that are freshly-drawn, 2 h, and 4 h after ambient air with 40% relative humidity. The hydrogel fibres contained 0.1 wt% VSNPs, without inserted twist or salt. The deformation rate was 1.1% s^{-1}. For (**b**) to (**d**), the hydrogel fibres were dried for 250 s, and the fibres contained 0.1 wt% VSNPs and 20 mM $ZnCl_2$, without inserted twist. The deformation rate was

1.1% s^{-1}. The relative humidity during test is 40%. For (**e**), the deformation rate was 27.8% s^{-1} and the twist density was 3 turns mm^{-1}. The relative humidity is 40%.

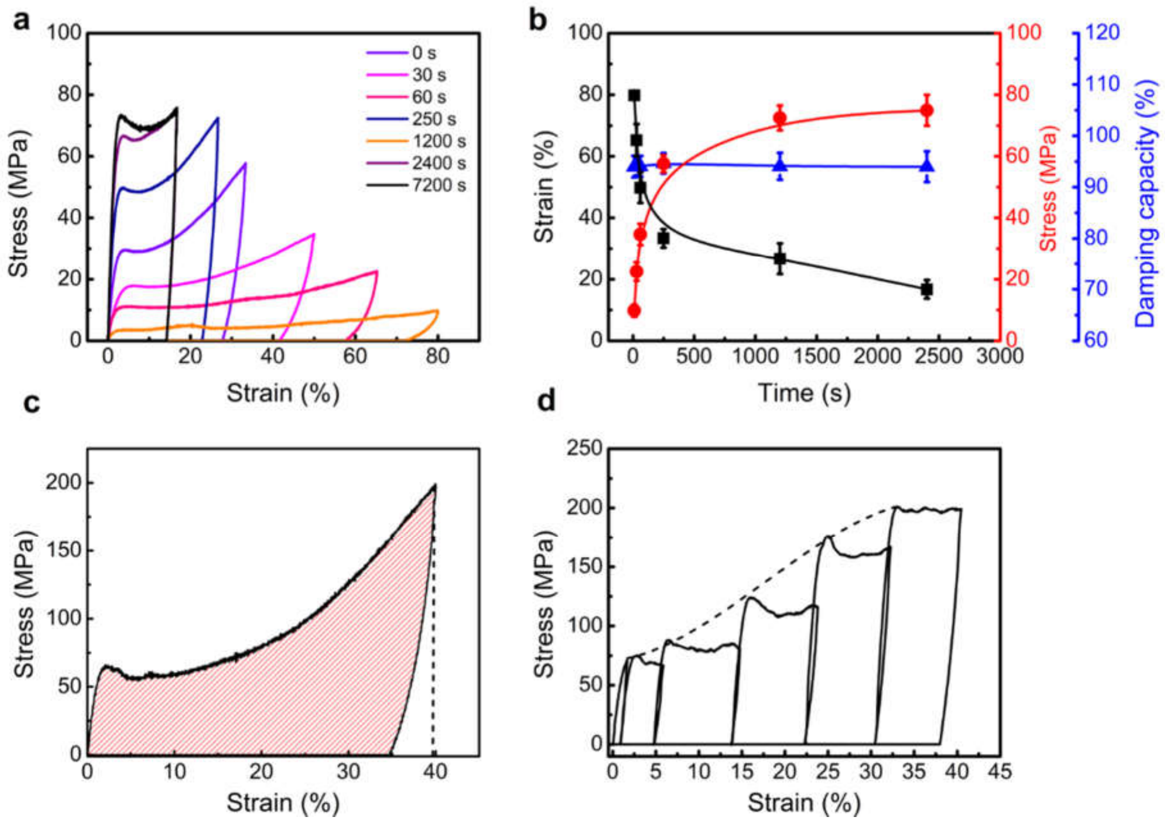

Supplementary Figure 11. (**a**) Loading-unloading curves of hydrogel fibres obtained after different drying times. (**b**) Stress, strain, and damping capacity of a hydrogel fibre as a function of drying time. For (**a**) and (**b**), the hydrogel fibres contained 0.1 wt% VSNPs, without inserted twist or salt, and the original diameter of fibres was 50 μm. The deformation rate was 1.1% s^{-1}. (**c**) Stress-strain curve of a hydrogel fibre containing 0.4 wt% VSNPs subjected to a loading-unloading cycle. The damping capacity was calculated using the ratio of the damping energy (the pink area encompassed by the loading and unloading curves) to the loading energy (the area encompassed by the loading curve and the dashed line). (**d**) Stress-strain curves of a hydrogel fibre containing 0.4 wt% VSNPs subjected to loading-unloading cycles with progressively increasing strain. The damping capacity amounted to 64% for the first cycle under an applied strain of 1.6% and increased to ~95% for subsequent cycles with applied strains from 5% to 40%. The hydrogel fibres contained 0.4 wt% VSNPs, without inserted twist or salt and the diameter of fibres was 20 μm. The deformation rate was 1.1% s^{-1}.

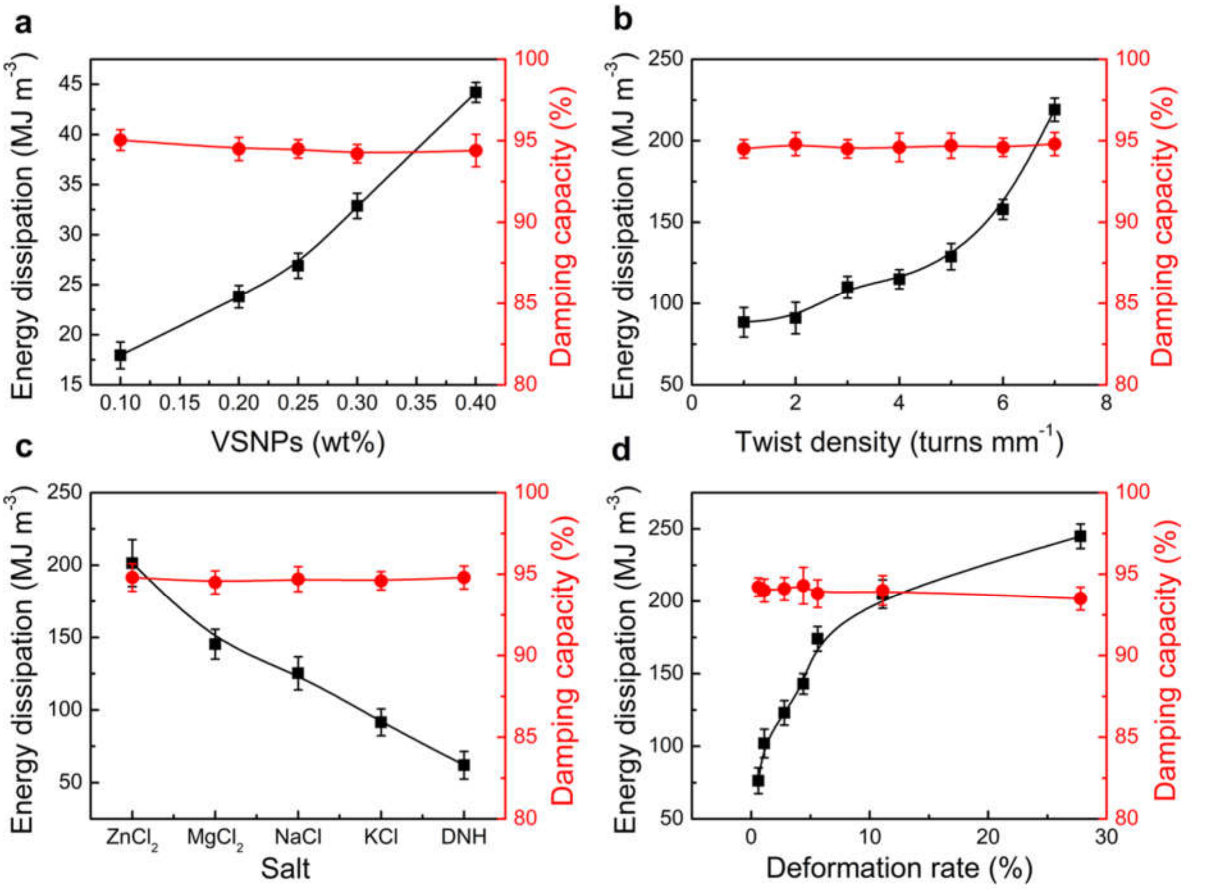

Supplementary Figure 12. Energy dissipation and damping capacity of a hydrogel fibre. (**a**) Dependence on VSNPs content, (**b**) twist insertion at a deformation rate of 27.8% s^{-1}, (**c**) salt composition, and (**d**) deformation rate. For (**a**), the diameter of fibres was 20 μm and the deformation rate was 1.1% s^{-1}. For (**b**), the hydrogel fibres contained 0.1 wt% VSNPs and 20 mM Zn^{2+}, and the diameter of fibres was 20 μm. The deformation rate was 27.8% s^{-1}. For (**c**), the hydrogel fibres contained 0.1 wt% VSNPs and 20 mM salt, and the diameter of fibres was 20 μm. The deformation rate was 27.8% s^{-1}, and the twist density was 3 turns mm^{-1}. For (**d**), the hydrogel fibres contained 0.1 wt% VSNPs and 20 mM Zn^{2+}, and the diameter of fibres was 20 μm. The twist density was 3 turns mm^{-1}.

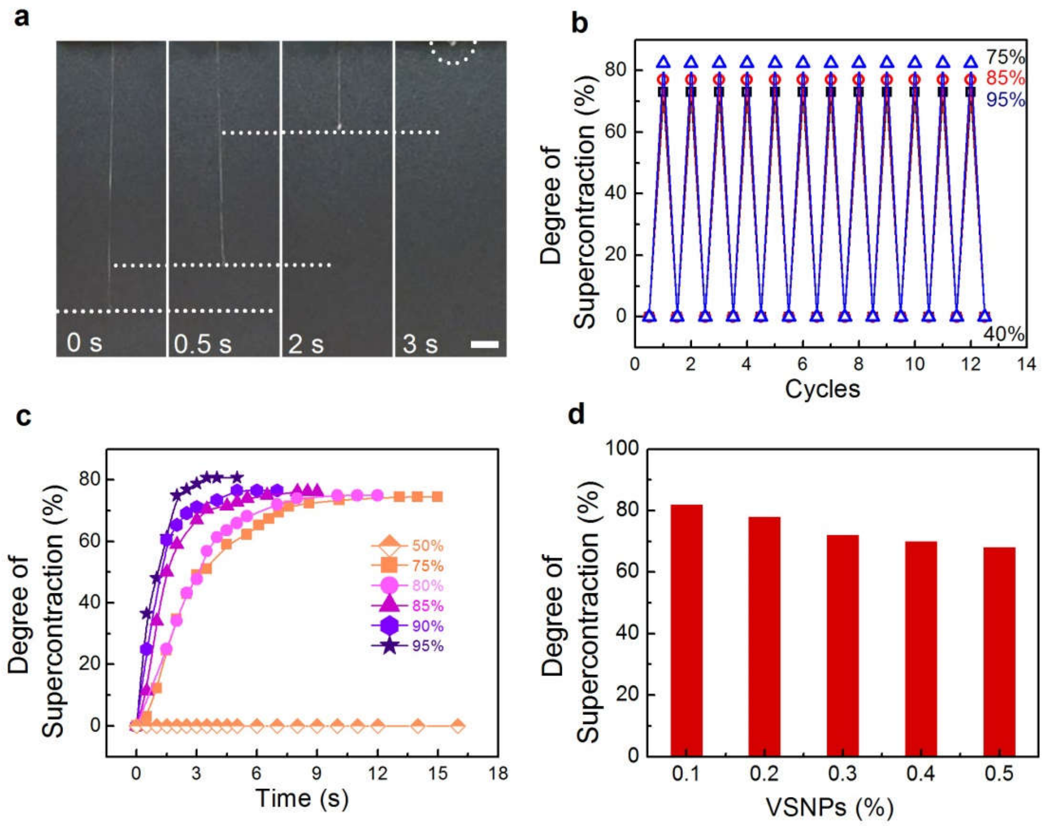

Supplementary Figure 13. (**a**) Snapshots of a 5-cm-long hydrogel fibre supercontracted into a 50-µm-diameter small ball at 95% RH. Scale bar: 1 cm. The white dashed lines indicate the supercontraction process of hydrogel fibres, and the white dashed oval presents the hydrogel small ball. (**b**) Supercontraction-stretching cycles obtained by switching between high and low RH. (**c**) Degree of supercontraction of a hydrogel fibre as a function of time at various RH. For (**a**) to (**c**), the hydrogel fibres contained 0.1 wt% VSNPs and 20 mM Zn^{2+}, and the diameter of fibres was 20 µm. (**d**) Degree of supercontraction of a hydrogel fibre as a function of VSNP content at 95% RH. For (**d**), the hydrogel fibres contained different VSNP content, without inserted twist or salt, and the diameter of fibres was 20 µm.

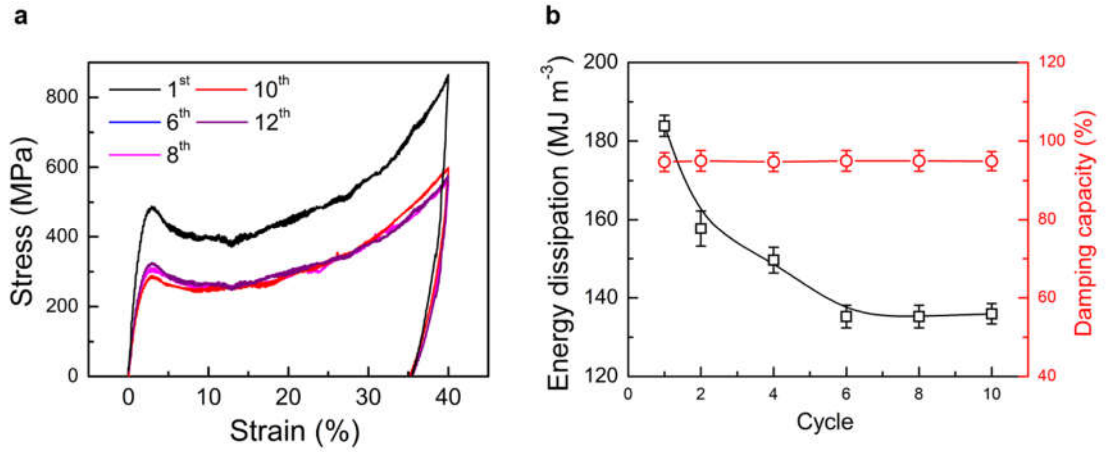

Supplementary Figure 14. (**a**) Stress-strain curves and (**b**) energy dissipation and damping capacity of a twisted hydrogel fibre during repeated loading-unloading cycles. The hydrogel fibre contained 0.1 wt% VSNPs and 20 mM $ZnCl_2$, with an inserted twist of 3 turns mm^{-1}, and the deformation rate was 27.8% s^{-1}.

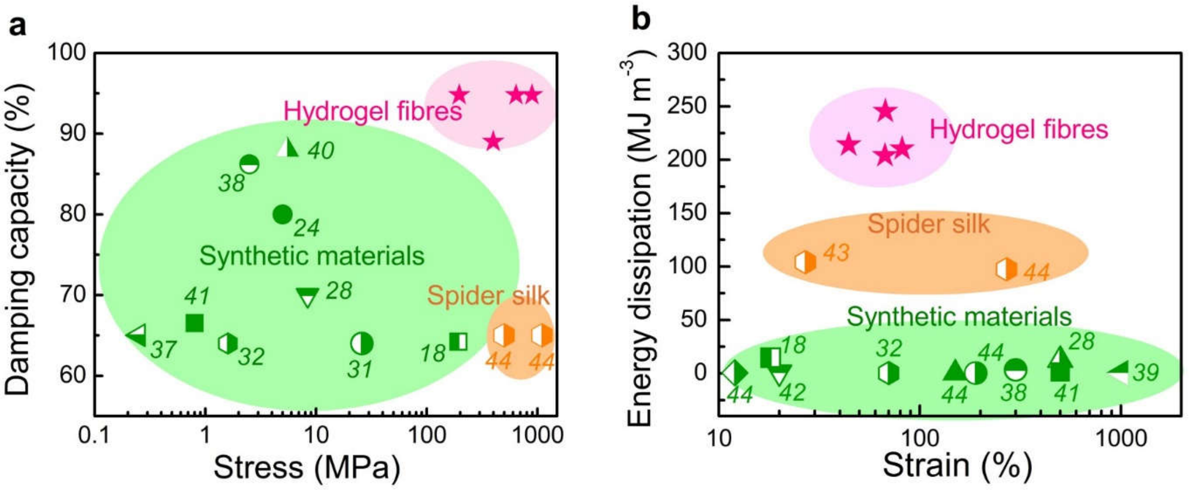

Supplementary Figure 15. (**a**) Comparison of stress and damping capacity of hydrogel fibres (pink stars) with those of other typical synthetic energy-dissipation materials (green symbols) and spider silks (yellow symbols). (**b**) Comparison of breaking stain and energy dissipation of hydrogel fibres (pink stars) with those of other typical energy-dissipation materials (green symbols) and spider silks (yellow symbols). The numbers shown in the graphs correspond to references in supporting information. The hydrogel fibres contained 20 mM $ZnCl_2$ with different inserted twists and deformation rates shown in Supplementary Tab. 3 and 4.

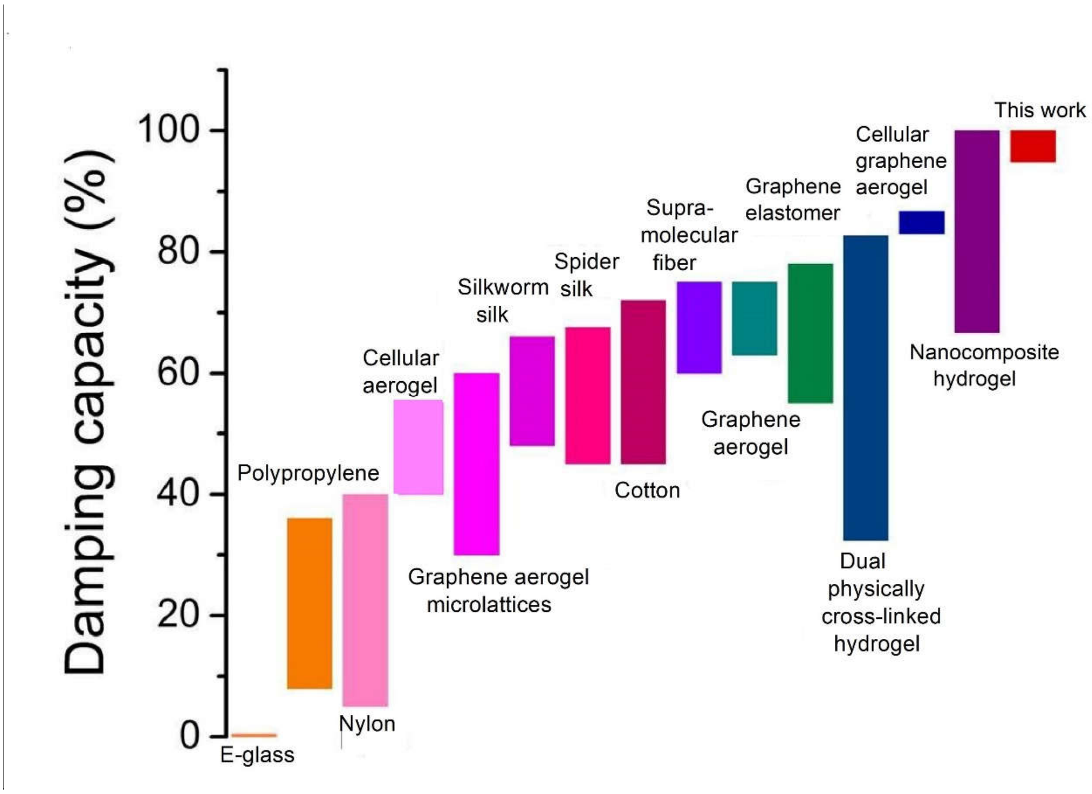

Supplementary Figure 16. Comparison of the damping capacity of hydrogel fibres with other typical energy-dissipation materials. These data are listed in Supplementary Tab. 5 and 6.

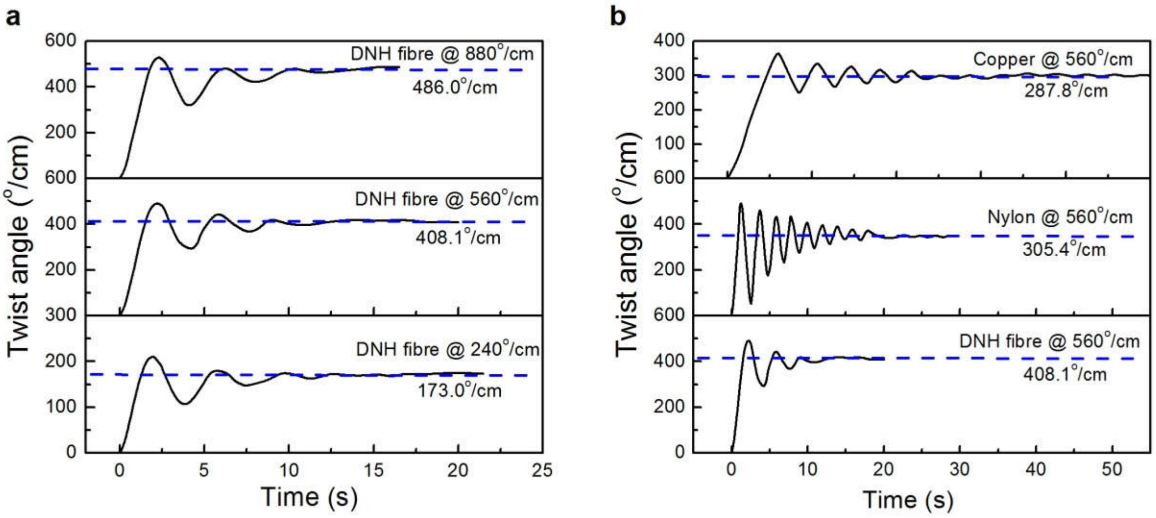

Supplementary Figure 17. (**a**) Torsional relaxation dynamics of hydrogel fibres containing 20 mM $ZnCl_2$ and different inserted twists. The blue dotted lines mark the new equilibrium positions. The applied load was 1.2 MPa and the moment of inertia of the hydrogel fibre was 8×10^{-15} kg m^2. (**b**) Torsional relaxation dynamics of a copper fibre, a nylon fibre, and a hydrogel fibre containing 20 mM $ZnCl_2$. The blue dotted lines mark the new equilibrium positions. The applied loads were 1.2, 63.6, and 3.9 MPa for the hydrogel, copper, and nylon fibres, respectively. The moments of inertia for the hydrogel, copper, and nylon fibres amounted to 8×10^{-15}, 8×10^{-13}, and 2.5×10^{-13} kg m^2, respectively.

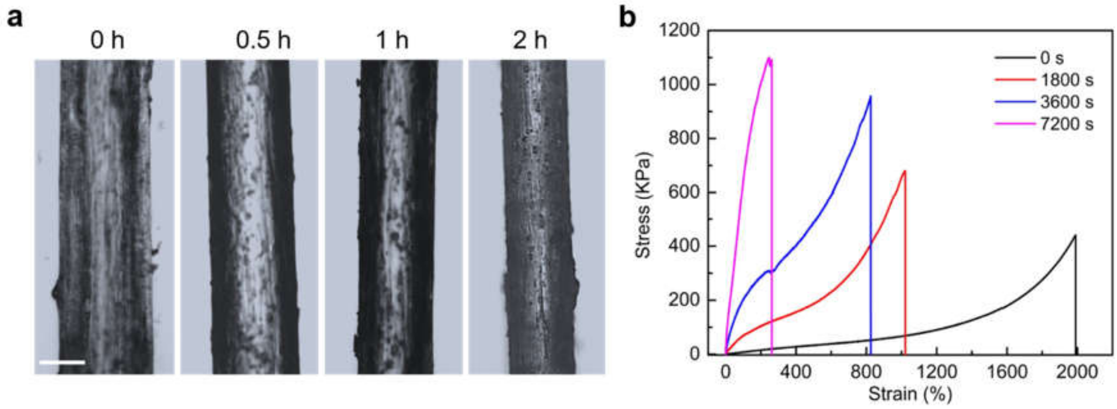

Supplementary Figure 18. (**a**) Metallographic microscopy images in reflective mode of polyacrylamide/alginate hydrogel fibre exposed in ambient air (40% humidity) for different time. Scale bar: 200 μm. The hydrogel is prepared according to literature[45], the fibre was prepared using a polypropylene tube (inner diameter of 0.40 mm, and outer diameter of 0.70 mm) as the template. (**b**) Stress-strain curves of the polyacrylamide/alginate hydrogel fibres in (a).

Supplementary References

1. Weatherbee-Martin, N. et al. Identification of wet-spinning and post-spin stretching methods amenable to recombinant spider aciniform silk. *Biomacromolecules* **17**, 2737–2746 (2016).
2. Adrianos, S. L. et al. Nephila clavipes Flagelliform silk-like GGX motifs contribute to extensibility and spacer motifs contribute to strength in synthetic spider silk fibers. *Biomacromolecules* **14**, 1751−1760 (2013).
3. Andersson, M. et al. Biomimetic spinning of artificial spider silk from a chimeric minispidroin. *Nat. Chem. Biol.* **13**, 262–264 (2017).
4. Yun, J. Y. et al. Production of a novel silk-like protein from sea anemone and fabrication of wet-spun and electrospun marine-derived silk fibers. *NPG Asia Mater.* **5**, 58–63 (2013).
5. Lin, Z., Deng, Q., Liu, X. Y., & Yang, D. Engineered large spider eggcase silk protein for strong artificial fibers. *Adv. Mater.* **25**, 1216–1220 (2013).
6. Seidel, A. et al. Regenerated spider silk: Processing, properties, and structure. *Macromolecules* **33**, 775−780 (2000).
7. Elices, M. et al. Bioinspired fibers follow the track of natural spider silk. *Macromolecules* **44**, 1166–1176 (2011).
8. You, Z. et al. Extraordinary mechanical properties of composite silk through hereditable transgenic silkworm expressing recombinant major ampullate Spidroin. *Sci. Rep.* **8**, 15956 (2018).
9. Peng, Q. et al. Recombinant spider silk from aqueous solutions via a bio-inspired microfluidic chip. *Sci. Rep.* **6**, 36473 (2016).
10. Aniela, H. et al. Biomimetic fibers made of recombinant spidroins with the same toughness as natural spider silk. *Adv. Mater.* **27**, 2189–2194 (2015).
11. Yan, J., Zhou, G., Knight, D. P., Shao, Z., & Chen, X. Wet-spinning of regenerated silk fiber from aqueous silk fibroin solution: discussion of spinning parameters. *Biomacromolecules* **11**, 1–5 (2010).
12. Zhou, G., Shao, Z., Knight, D. P., Yan, J. & Xin, C. Silk fibers extruded artificially from aqueous solutions of regenerated bombyx mori silk fibroin are tougher than their natural counterparts. *Adv. Mater.* **21**, 366–370 (2010).
13. Fang, G. et al. Insights into silk formation process: correlation of mechanical properties and structural evolution during artificial spinning of silk fibers. *ACS Biomater. Sci. Eng* **2**, 1992–2000 (2016).
14. Malay, A. D. et al. Relationships between physical properties and sequence in silkworm silks. *Sci. Rep.* **6**, 27573 (2016).
15. Zhang, Y., Yang, H., Shao, H. & Hu, X. Antheraea pernyi silk fiber: A potential resource for artificially biospinning spider dragline silk. *J. Biomed. Biotechnol.* **2010**, 1−8 (2010).
16. Anthoula, L. et al. Spider silk fibers spun from soluble recombinant silk produced in mammalian cells. *Science* **295**, 472–476 (2002).
17. Zhang, X. et al. Silkworms with spider silklike fibers using synthetic silkworm chow containing calcium lignosulfonate, carbon nanotubes, and graphene. *ACS Omega* **4**, 4832–4838 (2019).
18. Wu, Y. et al. Bioinspired supramolecular fibers drawn from a multiphase self-assembled

hydrogel. *Proc. Natl. Acad. Sci.* **114**, 8163–8168 (2017).

19. Shi, H. K. et al. Bio-inspired, moisture-powered hybrid carbon nanotube Yarn muscles. *Sci. Rep.* **6**, 23016 (2016).
20. Chen, P. et al. Hierarchically arranged helical fibre actuators driven by solvents and vapours. *Nat. Nanotechnol.* **10**, 1077–1083 (2015).
21. David, B., Mccarthy, D. N., Blau, W. J. & Coleman, J. N. Toughening of artificial silk by incorporation of carbon nanotubes. *Biomacromolecules* **8**, 3973–3976 (2007).
22. Steven, E. et al. Carbon nanotubes on a spider silk scaffold. *Nat. Commun.* **4**, 2435 (2013).
23. Filippidi, E. et al. Toughening elastomers using mussel-inspired iron-catechol complexes. *Science* **358**, 502–505 (2017).
24. Gui, X. et al. Three-dimensional carbon nanotube sponge-array architectures with high energy dissipation. *Adv. Mater.* **26**, 1248–1253 (2014).
25. Gao, H. L. et al. Super-elastic and fatigue resistant carbon material with lamellar multi-arch microstructure. *Nat. Commun.* **7**, 12920 (2016).
26. Roland, C. M. The mullins effect in crosslinked rubber. *J. Rheol.* **33**, 659 (1989).
27. Qiu, L., Liu, J. Z., Chang, S. L. Y., Wu, Y. & Li, D. Biomimetic superelastic graphene-based cellular monoliths. *Nat. Commun.* **3**, 1241 (2012).
28. Lin, P., Ma, S., Wang, X. & Zhou, F. Molecularly engineered dual-crosslinked hydrogel with ultrahigh mechanical strength, toughness, and good self-recovery. *Adv. Mater.* **27**, 2054–2059 (2015).
29. Gao, G., Du, G., Sun, Y. & Fu, J. Self-healable, tough, and ultrastretchable nanocomposite hydrogels based on reversible polyacrylamide/montmorillonite adsorption. *ACS Appl. Mater. Interfaces* **7**, 5029–5037 (2015).
30. Zhu, C. et al. Highly compressible 3D periodic graphene aerogel microlattices. *Nat. Commun.* **6**, 6962 (2015).
31. Cao, A. Y., Dickrell, P. L., Sawyer, W. G., Ghasemi-Nejhad, M. N. & Ajayan, P. M. Super-compressible foamlike carbon nanotube films. *Science* **310**, 1307–1310 (2005).
32. Zeng, Z. et al. Integrated random-aligned carbon nanotube layers: Deformation mechanism under compression. *Nanoscale* **6**, 1748–1755 (2014).
33. Song, G. et al. Facile fabrication of tough hydrogels physically cross-linked by strong cooperative hydrogen bonding. *Macromolecules* **46**, 7423–7435 (2013).
34. Sun, Y. N., Gao, G. R., Du, G. L., Cheng, Y. J. & Fu, J. Super tough, ultrastretchable, and thermoresponsive hydrogels with functionalized triblock copolymer micelles as macro-cross-linkers. *Acs Macro Letters* **3**, 496–500 (2014).
35. Bin Ihsan, A. et al. Self-healing behaviors of tough polyampholyte hydrogels. *Macromolecules* **49**, 4245–4252 (2016).
36. Chen, Q. et al. A novel design strategy for fully physically linked double network hydrogels with tough, fatigue resistant, and self-healing properties. *Adv. Funct. Mater.* **25**, 1598–1607 (2015).
37. Chen, Q., Zhu, L., Zhao, C., Wang, Q. & Zheng, J. A robust, one-pot synthesis of highly mechanical and recoverable double network hydrogels using thermoreversible sol-gel polysaccharide. *Adv. Mater.* **25**, 4171–4176 (2013).
38. Yang, Y., Wang, X., Yang, F., Shen, H. & Wu, D. A universal soaking strategy to convert composite hydrogels into extremely tough and rapidly recoverable double-network

hydrogels. *Adv. Mater.* **28**, 7178–7184 (2016).
39. Zhong, M., Shi, F. K., Liu, Y. T., Liu, X. Y. & Xie, X. M. Tough superabsorbent poly(acrylic acid) nanocomposite physical hydrogels fabricated by a dually cross-linked single network strategy. *Chin. Chem. Lett.* **27**, 312–316 (2016).
40. Yang, Y., Wang, X., Yang, F., Wang, L. & Wu, D. Highly elastic and ultratough hybrid ionic-covalent hydrogels with tunable structures and mechanics. *Adv. Mater.* **30**, 1707071 (2018).
41. Gong, Z. et al. High-strength, tough, fatigue resistant, and self-healing hydrogel based on dual physically cross-linked network. *ACS Appl. Mater. Interfaces* **8**, 24030–24037 (2016).
42. Mredha, M. T. I. et al. A facile method to fabricate anisotropic hydrogels with perfectly aligned hierarchical fibrous structures. *Adv. Mater.* **30**, 1704937 (2018).
43. Elices, M., Plaza, G. R., Pérez-Rigueiro, J. & Guinea, G. V. The hidden link between supercontraction and mechanical behavior of spider silks. *J. Mech. Behav. Biomed. Mater.* **4**, 658–669 (2011).
44. Gosline, J. M., Guerette, P. A., Ortlepp, C. S. & Savage, K. N. The mechanical design of spider silks: From fibroin sequence to mechanical function. *J. Exp. Biol.* **202**, 3295–3303 (1999).
45. Sun, J.-Y. *et al.* Highly stretchable and tough hydrogels. *Nature* **489**, 133–136 (2012).
