# Supplementary material for: Artificial spider silk from ion-doped and twisted core-sheath hydrogel fibres
Source: Nat Commun. 2019 Nov 22;10:5293. doi: 10.1038/s41467-019-13257-4 (PMC6874677; doi:10.1038/s41467-019-13257-4)
Supplement: Supplementary file 3 — Description of Additional Supplementary Files [file 41467_2019_13257_MOESM3_ESM.pdf]

Description of Additional Supplementary Files

File name: Supplementary Movie 1

Description: Energy dissipation and impact force reduction of a hydrogel yarn and a cotton yarn with a vertical configuration.

File name: Supplementary Movie 2

Description: Energy dissipation and impact force reduction of a hydrogel yarn, a cotton yarn, and a rubber fibre with a horizontal configuration.

File name: Supplementary Movie 3

Description: Energy dissipation of shock-absorbing nets knitted with hydrogel yarns and cotton yarns. An egg free-falling from one metre was caught undamaged by the hydrogel net, but an egg broke when captured by the cotton web. The hydrogel yarn consisted of 30-cm-long, 100-ply, 20-μm-diameter hydrogel fibres with an inserted twist of 3 turns mm^{-1}. The cotton yarn measured 10-cm-long and 200 μm in diameter.

File name: Supplementary Movie 4

Description: A web knitted with hydrogel yarn that becomes deformed under a 200-g load retrieves its initial shape via supercontraction at 60% RH. The hydrogel yarn consisted of 30-cm-long, 100-ply, 20-μm-diameter hydrogel fibres with an inserted twist of 3 turns mm^{-1}. The net was placed into a chamber at 60% humidity, then the weight was placed; after the hydrogel net deformed to a stable state, the weight was removed. During this process, the humidity was kept at 60%. A running timer was added into the video to displaying elapsed time.
